# Impact of fixation duration on messenger RNA detectability in human formalin-fixed paraffin-embedded brain tissue

**DOI:** 10.1093/braincomms/fcae430

**Published:** 2024-11-28

**Authors:** Charlene-Annett Hurler, Sabine Liebscher, Thomas Arzberger, Sarah Jäkel

**Affiliations:** Institute for Stroke and Dementia Research, LMU University Hospital, Ludwig-Maximilians-University Munich, 81377 Munich, Germany; Institute of Clinical Neuroimmunology, LMU University Hospital, Ludwig-Maximilians-University Munich, 82152 Planegg-Martinsried, Germany; Biomedical Center (BMC), Faculty of Medicine, Ludwig-Maximilians-University Munich, 82152 Planegg-Martinsried, Germany; Department of Neurology, University of Cologne & University Hospital of Cologne, 50937 Cologne, Germany; Munich Cluster for Systems Neurology (SyNergy), 81377 Munich, Germany; Department of Psychiatry and Psychotherapy, University Hospital, Ludwig-Maximilians-University Munich, 80336 Munich, Germany; Center for Neuropathology and Prion Research, Ludwig-Maximilians-University Munich, 81377 Munich, Germany; Institute for Stroke and Dementia Research, LMU University Hospital, Ludwig-Maximilians-University Munich, 81377 Munich, Germany; Munich Cluster for Systems Neurology (SyNergy), 81377 Munich, Germany

**Keywords:** human FFPE tissue, post-mortem brain tissue, transcriptomic approaches, RNAscope, mRNA

## Abstract

Technologies to study mRNA in post-mortem human brain samples have greatly advanced our understanding of brain pathologies. With ongoing improvements, particularly in formalin-fixed paraffin-embedded tissue, these technologies will continue to enhance our knowledge in the future. Despite various considerations for tissue and mRNA quality, such as pre-mortem health status and RNA integrity, the impact of the tissue fixation time has not been addressed in a systemic fashion yet. In this study, we employed RNAscope to assess mRNA detectability in human post-mortem brain tissue in relation to fixation time. Our results reveal a dynamic change in mRNA detection across varying fixation durations, accompanied by an increase in signal derived from the negative probe and autofluorescence background. These findings highlight the critical relevance of standardized fixation protocols for the collection of human brain tissue in order to probe mRNA abundancy to ensure reliable and comparable results.

## Teaser

Hurler *et al*. performed RNAscope on post-mortem human brain tissue and found mRNA signal detection to be highly dependent on fixation time. Considering the growing interest in the usage of human tissue in research, this finding highlights the importance of standardized protocols for the collection and preservation of such specimens.

## Graphical Abstract

**Graphical Abstract:**
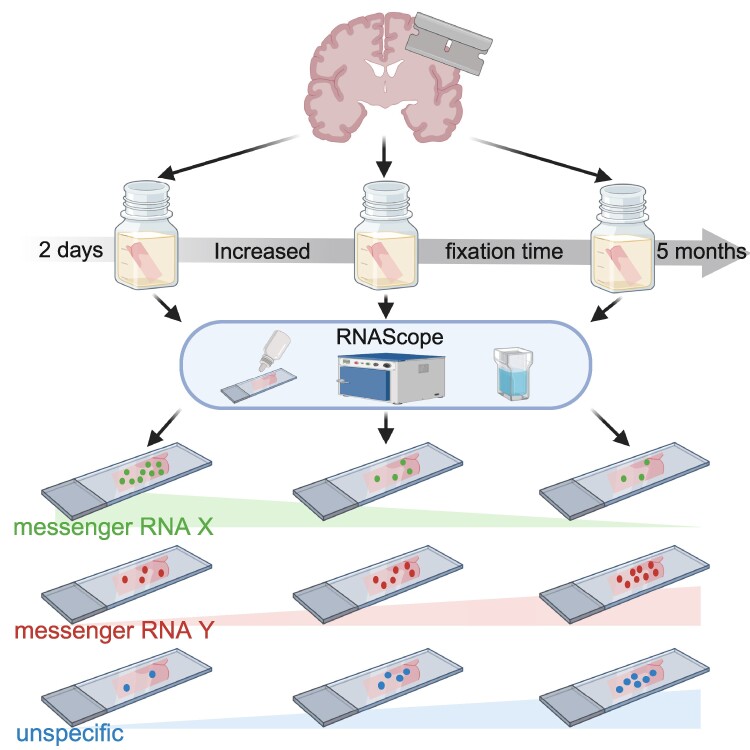

## Introduction

The conservation of post-mortem human brain material for pathological, anatomical and molecular investigation has a long history.^1^ However, due to technological advancements in genetic modification and higher reproducibility, concomitant with the lack of high-quality human tissue, animal models have mostly replaced the use of human brain tissue in science.^2-4^

Single-cell and spatial transcriptomic technologies to study fundamental and molecular disease mechanisms have exponentially increased^5^ and have more recently also tremendously improved our understanding of human neurological disorders,^6-8^ which has in turn led to an enhanced demand for human patient material. Due to the limited availability of fresh autopsy material, especially in large cohort studies, the use of archived formalin-fixed paraffin-embedded (FFPE) material provided by brain banks has become common practice. With this, also the single-nucleus RNA-seq technology has proven highly efficient.^9^ Whilst snRNA-seq studies mostly rely on fresh-frozen material, spatial transcriptomic or other *in situ* hybridization-based techniques that are using FFPE material are currently on the rise.^10-12^ This offers the advantage of being more frequently available, of higher tissue stability with conserved cellular tissue morphology, especially for methods that require extensive pre-treatment, as well as easier storage possibilities. Although high-resolution transcriptomic technologies are continuously improving in terms of spatial resolution and sequencing depth, the quality of post-mortem human tissue samples is mostly inferior in comparison to mouse tissue and thus often results in mRNA degradation, hampering mRNA detection^13,14^ and causing highly variable results.

Many variables determining the quality of post-mortem brain tissue have been discussed at length, including the pre-mortem health condition of the donor, tissue or ventricular fluid pH and the interval between time of death and tissue preservation.^15-18^ However, there is no consensus or definition of what can be considered good-quality tissue. Regarding mRNA preservation, the RNA integrity number (RIN) value is currently used as standard, despite considerable doubts regarding its predictability of overall tissue quality.^19,20^ Few studies have revealed that fixation time of tissue samples also influences the RNA quality in terms of RIN values and RT-PCR results on a global level,^21,22^ but too little attention is devoted to this variable during tissue collection and most brain banks do not have standardized protocols, when it comes to fixation of post-mortem samples.

Here, we aim to assess the effect of fixation time of post-mortem human brain tissue samples on the detectability of individual mRNAs by RNAscope *in situ* hybridization in order to work towards a standardized protocol for the collection of post-mortem human brain tissue. We developed a workflow to fix already archived frozen brain tissue, without destroying tissue integrity, and assessed mRNA detection after different fixation times. Applying RNAscope *in situ* hybridization, our work demonstrates a decline in the reliable detection of mRNA puncta with a concomitant increase in background signal and unspecific binding with increasing fixation time. As the use of human FFPE brain tissue is becoming increasingly important, the results of our study suggest the implementation of standardized protocols for tissue fixation across brain banks to foster reliable results and allow for comparability of results across different studies and tissue banks.

## Materials and methods

### Human brain donors

Tissue from three different brain donors was obtained from the Neurobiobank Munich. Details about the human donors are reported in Table 1.

**Table 1:** Human donor information

Donor ID	Sex	Age (years)	PMI (h)
#01	Male	19	18
#02	Female	46	15−18
#03	Female	80	12

### Tissue fixation

Large tissue blocks (∼2 cm × 2 cm × 1 cm) from three different donors from archived, fresh snap-frozen tissue from the same brain region (occipital cortex) were thawed at RT for ∼15 min until the tissue surface became shiny and subsequently separated into five equally sized pieces, which were immediately transferred into 4% neutral phosphate-buffered formaldehyde. For each donor, the five tissue blocks were fixed at five different timepoints: 2 days, 5 days, 2 weeks, 2 months and 5 months. Tissue blocks were consecutively dehydrated in a descending ethanol row and Roti-Histol for a total of 68 h in an automated carousel and embedded in paraffin, and 4 µm sections were cut at a microtome.

### RNAscope

Fluorescent Multiplex assay (Bio-techne ACD) of FFPE tissue samples has been performed, according to the manufacturer’s instructions: 4 µm FFPE sections were baked at 60°C for 1 h, followed by deparaffinization in 100% xylol twice for 5 min each and in 100% ethanol twice for 2 min each.

Tissue pre-treatment was performed by boiling the sections for 15 min in Target Retrieval Reagent (322000, Bio-techne ACD) using a steamer. A hydrophobic barrier was then drawn around each tissue section with the ImmEdge Pen (H-4000), followed by 30-min incubation with Protease Plus at 40°C (322331). Probes were hybridized at 40°C for 2 h, and signal enhancement was carried out using the RNAscope V2 Multiplex Fluorescent Detection Kit (323110, Bio-techne ACD). The colour reaction was performed using TSA Vivid dyes 570 and 650 (1:1500, 7526/1, 7527/1, Tocris). The following catalogue probes were used: Hs-ALDH1L1-C2 (438881), Hs-TMEM119-C3 (478911-C2), Hs-SCNA-C1 (605681-C3) and Hs-OLIG2-C2 (424191-C2), negative probe (320871).

### Imaging and quantifications

Images (332.8 × 332.8 µm, 2048 × 2048 pixels) were randomly taken with a Zeiss Axio Observer Z1 with a ×40 objective within a short time after the experiment to avoid fading of the signal. From each cortical region (upper, middle and deep layers) and each donor and timepoint, six random images, selected on the DAPI channel, were taken and averaged in the analysis. Where possible, mRNA detection was performed automated using CellProfiler image analysis software.^23^ In the CellProfiler pipeline, multichannel images were separated and analysed separately with the following parameters: typical diameter of positive signal: 2–20 pixel units; threshold strategy to separate background: global, minimal cross-entropy with 0.08 as lower and 1.0 as upper bound of the threshold. In cases where autofluorescence background was brighter than the mRNA signal, mRNA quantification was performed semi-automated, using Fiji default automated thresholding (positive signal detection purely based on intensity) or manually using the CellCounter plugins in the Fiji imaging software.^24^ In the manual scenario, mRNA puncta were only counted ‘positive’ when the signal was a round dot and when there was no overlap in the signal in other fluorescent channels; otherwise, it was considered autofluorescence background. In order to rule out analytical bias, individual images were randomized for mRNA detection using a Fiji randomization plugin and quantification was carried out blinded.

For the quantification of the puncta per positive cell first, the number of positive cells over all DAPI cells was determined. Only cells with three or more puncta on or closely around the nucleus were counted as positive. After this, the number of puncta concentrated in and around the nucleus was determined.

To determine the autofluorescence background, we acquired images of the green fluorescent channel in the absence of a green fluorescent probe, as background fluorescence is typically highest in that spectral range. Quantification of the autofluorescence background was done using the Fiji imaging software: randomized and blinded images (6–10 images per donor and region) were thresholded for the positive signal, using the default thresholding in the Fiji plugin and the percentage area of positive signal in the image was measured.

For visualization purposes in the figures, fluorescent mRNA signal was inverted into black and white images using the Fiji ‘invert’-option on 16-bit colour images.

### Statistical analysis

Statistical analysis was performed using GraphPad Prism software 10.0.3. All *P*-values were determined using an unpaired *t*-test (two groups) or an ordinary one-way ANOVA with Tukey’s multiple comparison *post hoc* test (multiple groups): *P* > 0.05 not significant, *P < 0.05, **P < 0.01, and ***P < 0.001.

### Ethics approval and consent to participate

The use of human brain tissue in this study has been approved by the ethics committee at the Ludwig-Maximilians University in Munich, ethical vote 20-1002. Tissue samples were provided by the Neurobiobank Munich, Germany.

## Results

### Fixation time negatively influences the detectability of mRNAs and increases autofluorescence background

**Figure 1:**
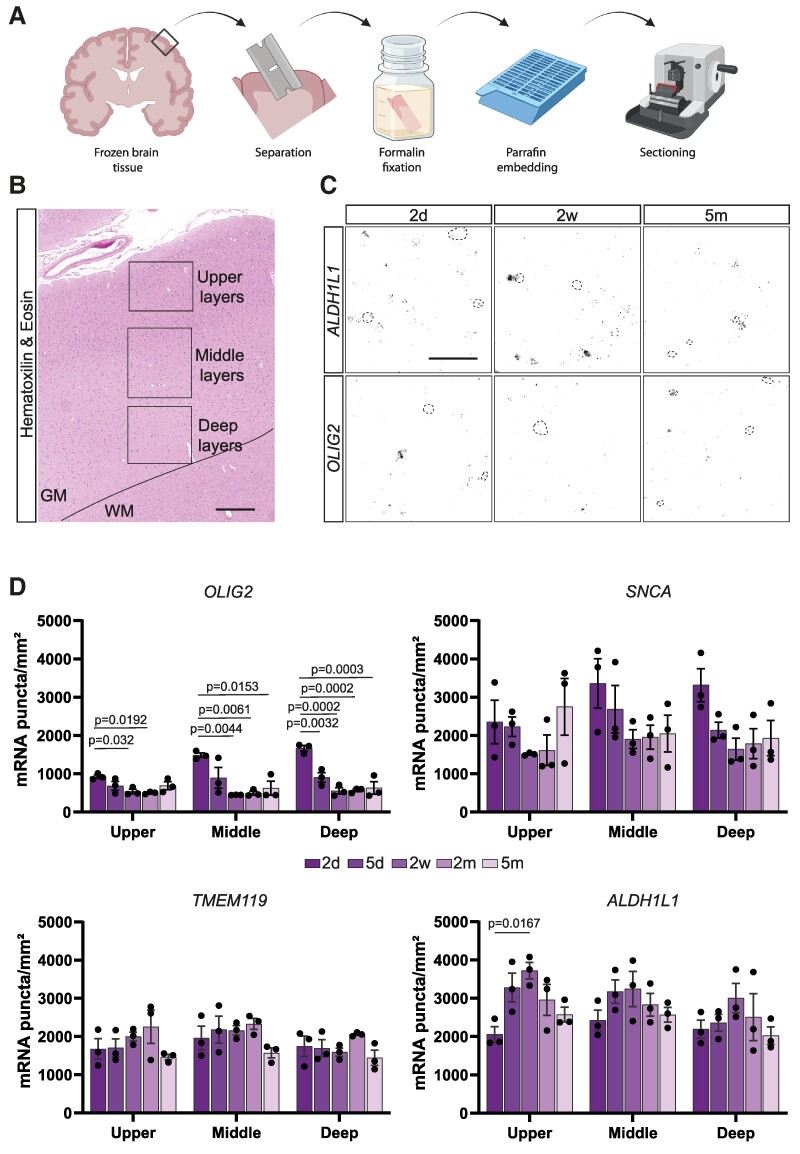
**Detection of mRNA significantly changes with fixation time.** (**A**) Scheme of experimental procedure for tissue collection, fixation and embedding. (**B**) Overview of the cortical regions that were selected for image quantification. Scale bar = 500 µm. (**C**) Representative images from donor #02 of *OLIG2* and *ALDH1L1* mRNA puncta in the middle cortical layers at different timepoints after fixation. Dashed outlines represent background signal that was not considered for quantification. Scale bar = 50 µm. (**D**) Quantifications of *OLIG2*, *SNCA*, *TMEM119* and *ALDH1L1* mRNA puncta across upper, middle and deep cortical layers at different timepoints after fixation in each donor. The detection of mRNA shows significant variability with prolonged fixation. Data represented as mean ± SEM, and each data point represents the average of an individual donor (*n* = 3). An ordinary one-way ANOVA with Tukey’s multiple comparison *post hoc* test was used for statistical analysis: *OLIG2*: upper layers: *F* = 4.755, middle layers: *F* = 8.071, deep layers: *F* = 20.02. *ALDH1L1*: upper layers: *F* = 4.728, middle layers: *F* = 1.301, deep layers: *F* = 1.048. *SNCA*: upper layers: *F* = 1.233, middle layers: *F* = 1.640, deep layers: *F* = 3.315. *TMEM119*: upper layers: *F* = 1.401, middle layers: *F* = 1.572, deep layers: *F* = 1.504. Individual significant *P*-values are displayed in the figure; other comparisons did not show significance. **A** was created with *Biorender.com*.

For this study, we used a large snap-frozen tissue block from each donor that was separated into five equally sized smaller blocks and fixed in 4% neutral-buffered formaldehyde. The blocks were collected at five different timepoints: 2 days, 5 days, 2 weeks, 2 months and 5 months (Fig. 1A). Our initial concern was whether freeze-thawing would compromise its integrity and structural components; however, our histological analysis, comparing previously FFPE sections of initially snap-frozen samples with primarily immersion-fixed tissue sections, revealed no overt discernible difference in tissue quality and integration (Supplementary Fig. 1A).

We then performed RNAscope *in situ* hybridization on our tissue sections across the different timepoints of fixation and quantified individual mRNAs within the cortex in order to determine how the fixation time influences mRNA detection (Fig. 1; Supplementary Fig. 1B). We selected probes for mRNAs that can be attributed to the four major cell types in the brain: *OLIG2* for oligodendrocytes, *SNCA* for neurons, *TMEM119* for microglia and *ALDH1L1* for astrocytes. As not all cell types are equally distributed within the cortex, which holds especially true for oligodendrocytes and neurons,^25,26^ we have separated our analysis by cortical layers: upper, middle and deep layers (Fig. 1B). *OLIG2* and *SNCA* mRNA as well as *TMEM119* and *ALDH1L1* mRNA were performed together on the same tissue section in different channels, and the experiments were performed in larger experimental batches, which always included all donors and at least two fixation times to rule out technical batch effects.

Overall, there were substantial differences in the number of mRNA puncta detectable across the different timepoints; however, not all mRNAs were affected equally (Fig. 1C and D; Supplementary Fig. 1B). *OLIG2* mRNA was already reduced at 5 days in comparison to the 2-day timepoint and remained at that detection level throughout all further timepoints. Although not statistically significant, there was also a clear trend towards lower mRNA counts at longer fixation times for *SNCA*. *TMEM119* mRNA, on the other hand, was barely affected by the different fixation times. Surprisingly, *ALDH1L1* mRNA significantly increased with fixation time (Fig. 1D). Whilst the fixation time seems highly variable across different mRNAs, the dynamics of all mRNAs followed a very similar pattern (decrease or increase) in the different cortical layers.

**Figure 2:**
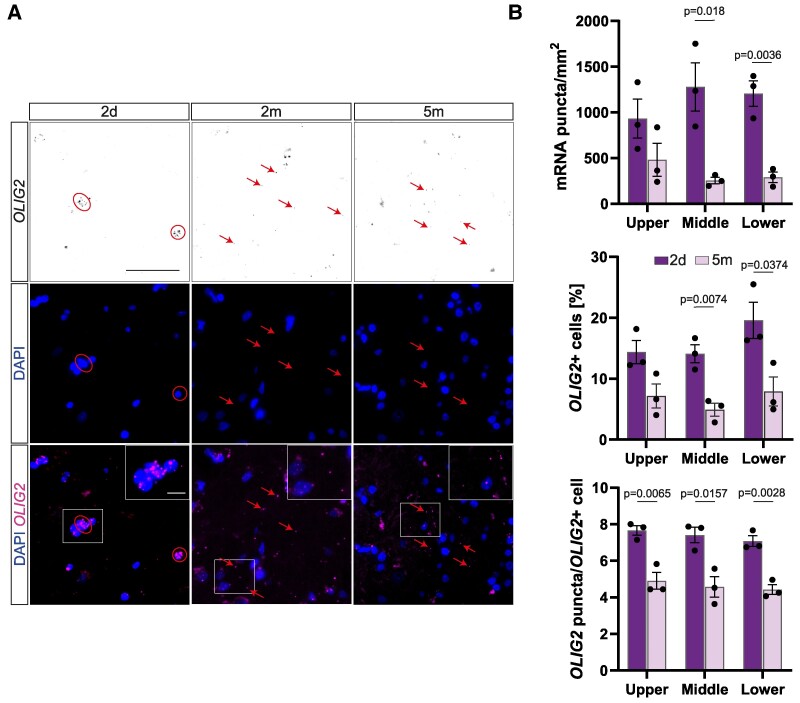
**Prolonged fixation reduces the number of *OLIG2*+ cells and *OLIG2* mRNAs within a cell.** (**A**) Examples from donor #02 of how spatial *OLIG2* mRNA distribution changes with increased fixation time. At 2-day fixation, *OLIG2* mRNA is mostly located around the DAPI+ nucleus (circled area) whilst at 2 and 5 months, the mRNA signal is more dispersed in the parenchyma (arrows) indicating an increase in unspecific binding. Scale bar = 50 µm. (**B**) Quantifications of *OLIG2* mRNA puncta in close proximity to the nucleus (upper panel), the percentage of *OLIG2*+ cells (middle panel) and the average mRNA puncta per *OLIG2*+ cell at 2 days and 5 months of fixation in different cortical layers. The quantifications show a significant decrease, further indicating a loss of the specific signal. Data represented as mean ± SEM, and each data point represents the average of an individual donor (*n* = 3). An unpaired two-tailed *t*-test was used for statistical analyses. mRNA puncta/mm^2^: upper layers: *t* = 1.611, middle layers: *t* = 3.869, deep layers: *t* = 6.106. % *OLIG2*+ cells: upper layers: *t* = 2.620, middle layers: *t* = 5.026, deep layers: *t* = 3.068. *OLIG2* puncta/*OLIG2*+ cell: upper layers: *t* = 5.201, middle layers: *t* = 4.033, deep layers: *t* = 6.579. Individual significant *P*-values are displayed in the figure; other comparisons did not show significance.

At 2 days of fixation, the *OLIG2* and *SNCA* mRNA distribution followed the expected laminar patterns: oligodendrocytes and neurons are sparser in upper cortical layers and denser in deeper ones.^25,26^ Although we detected higher levels of mRNA after longer fixation times, the cortical layer patterning of oligodendrocytes and neurons was already absent after 5 days of fixation (Fig. 1D), strongly suggesting false positive signal, accompanied by loss of real signal Moreover, when assessing the spatial distribution of mRNA puncta across timepoints for the *OLIG2* mRNA, we noticed a profound difference: Whilst at 2-day fixation *OLIG2* mRNA was mostly concentrated in and around DAPI+ nuclei, which would be the expected localization, it was more dispersed without an associated nucleus at later timepoints, further indicating unspecific binding in the tissue (Fig. 2A). In order to measure this phenomenon, we re-quantified *OLIG2* mRNA at 2 days and 5 months of fixation and only quantified puncta localized in close proximity to DAPI+ nuclei and similarly found a significant reduction in the total number of puncta, in the number of individual mRNAs per *OLIG2*+ cell and in the proportion of *OLIG2*+ cells (Fig. 2B).

**Figure 3:**
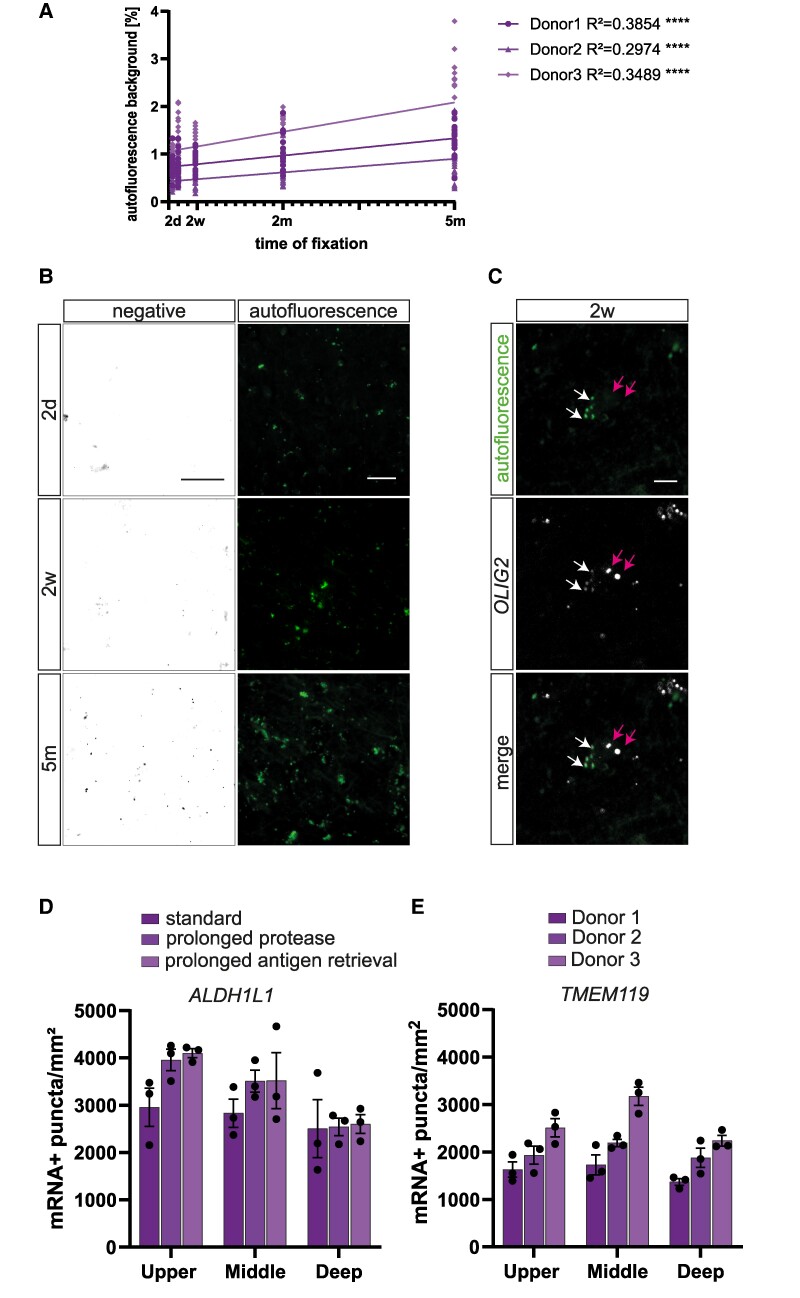
**Prolonged fixation increases unspecific signal.** (**A**) Quantification of the autofluorescence signal in the green channel without mRNA probe across different fixation times shows a significant increase in each patient. Individual data points represent single images (*n* = 15–18 images for each of the 3 donors at each timepoint). No statistical tests were applied. (**B**) Examples of increased signal in middle cortical layers in the negative probe (from donor #01) and the autofluorescence background (from donor #03) between 2 days, 2 weeks and 5 months of fixation. Scale bars = 25 µm (negative) and 50 µm (autofluorescence). (**C**) High-resolution images of autofluorescence signal that closely resembles *OLIG2* mRNA signal (white arrows) next to real mRNA signal (magenta arrows) at 2 weeks of fixation from donor #01. Scale bar = 10 µm. (**D**) Quantification of *ALDH1L1* mRNA with standard pre-treatment conditions, prolonged protease and prolonged boiling, showing a tendential increase in detected mRNA after 2 months of fixation. Data represented as mean ± SEM, and each data point represents the average of an individual donor (*n* = 3). An ordinary one-way ANOVA with Tukey’s multiple comparison *post hoc* test was used for statistical analysis: Upper layers: *F* = 5.127, middle layers: *F* = 0.9598, deep layers: *F* = 0.01601. No comparison did show a significant *P*-value. (**E**) Quantification of *TMEM119* mRNA at 5 days of fixation after repetition of the experiment on the same donor in three independent batches demonstrating a low degree of variation and thus high reproducibility. Data represented as mean ± SEM, and individual data point represents the average of one experimental batch (*n* = 3). No statistical tests were applied.

Human brain tissue is known to have strong autofluorescence, especially in the green spectral range, rendering analysis of fluorescent images sometimes challenging, especially when the true positive signal is as small as mRNA puncta. In order to determine whether the fixation time also influences the autofluorescence background of the tissue, we quantified the signal intensity in the green fluorescent channel in the absence of an RNA probe reflecting autofluorescence. Indeed, the area in the pictures that was covered by autofluorescence background significantly increased with fixation time in each donor (Fig. 3A and B). Especially at long fixation times, the autofluorescence signal increasingly matched the actual RNAscope mRNA signal, also in signal intensity, making it more difficult to distinguish between autofluorescence background and real mRNA signal (Fig. 3C).

In order to address whether longer fixation causes more unspecific binding of mRNA probes, we used an RNAscope negative probe, targeting a bacterial gene (*dapB*), which is not expressed in the human brain and indeed found an increase in positive signal with longer fixation for each patient (Fig. 3B).

Taken together, our experiments demonstrate that mRNA detection significantly changes with prolonged fixation times. This seems to be caused by a combination of decreased specific detection of mRNA molecules, going hand in hand with unspecific binding, and an increase in the autofluorescence background, which can often be misinterpreted as real signal. These alterations led to a disruption of the known laminar spatial distribution of cell type-specific mRNAs upon prolonged fixation times and argue for an unreliable signal.

### Longer antigen retrieval does not improve mRNA accessibility in over-fixed tissue

Formalin fixation preserves tissue integrity by cross-linking proteins, yet it concurrently obscures intracellular mRNAs. To overcome this, the RNAscope procedure, like other mRNA detection protocols, incorporates two critical steps to make the mRNA more accessible. In this case, this includes treatment of the tissue with protease and antigen retrieval by boiling of the tissue in a steamer in target retrieval reagent. We investigated if employing extended and harsher pre-treatment conditions could change mRNA accessibility in tissues subjected to prolonged fixation. We therefore repeated mRNA detection for *ALDH1L1* and *TMEM119* mRNA in the 2-month fixed tissue blocks and extended (i) the protease digestion from 30 to 40 min (prolonged protease) and (ii) the antigen retrieval step from 15 to 25 min (prolonged antigen retrieval) and compared it to the standard protocol (Fig. 3D; Supplementary Fig. 1C). Whereas the type of the pre-treatment (protease or antigen retrieval) did not make a difference in the detectability of the mRNA in these two examples, for *ALDH1L1*, there was a substantial (roughly 30%) increase in the upper layers of the cortex in comparison to the standard treatment. Given our previous results, that longer fixation increases unspecific binding, the additional increase in signal detection might also not be specific.

In brief, extended tissue pre-treatment can change mRNA detectability; however, very likely this increase is caused by unspecific binding of the probes and might not be beneficial.

### Detection of mRNA at short fixation times is reproducible

Human brain tissue is precious and RNA detection methods are expensive, wherefore often, such experiments are only performed with one technical replicate. For this reason, we lastly wanted to address the reproducibility of our results and repeated the detection of *TMEM119* mRNA after 5 days of fixation twice more in each patient in independent experimental batches (Fig. 3E) and compared the results from each experimental batch. The average deviation from the mean was 13% (ranging between 6 and 20%), demonstrating that overall, the results were quite reproducible across the different experimental batches and donors.

## Discussion

Our study systematically investigated the impact of fixation time on the detectability of mRNAs by RNAscope in defrosted and consecutively fixed post-mortem human brain tissue blocks. We first show that the fixation of the previously snap-frozen tissue does not negatively influence tissue quality. This is a crucial finding as it underscores the feasibility of using snap-frozen tissue, which is more versatile for future experimental procedures and at the same time decreases technical bias. Slowly frozen tissue, on the other hand, might not be suitable for this purpose, as tissue morphology is generally not maintained and a lot of freezing artefacts are visible. Some mRNA detection methods might work well with non-embedded frozen tissue; however, due to the many repetitive washing and reagent incubation steps, the tissue is easily destroyed. Therefore, FFPE sections are preferable for most mRNA detection methods, with the advantage that the tissue is preserved well enough that the *in situ* hybridization can be combined with immunohistochemistry on the same section after the mRNA detection procedure.

We have further demonstrated significant variability in the detectability of different mRNAs using the RNAscope technology in relation to fixation time. After repetition, our results were also reproducible, although there is currently no general consensus about the expected variation. Although this study was a proof of principle, utilizing only three human brain donor samples and four different mRNA probes, it already revealed a clear correlation between fixation time and mRNA detectability. This relationship is not straightforward, as mRNA puncta do not simply decrease linearly with fixation time; instead, ‘detectability’ varies depending on the individual mRNA, and even an increase is possible (as seen for *ALDH1L1*). However, due to the mRNA’s distribution, which is contrary to the biologically expected distribution both in the cortical layers and in its concentration around nuclei (as shown for *OLIG2*), we suspect that this increase in mRNA quantity mostly reflects non-specific binding rather than genuine signal. This suspicion is further supported by the increase in detected signal from the negative probe and the rise in autofluorescence; therefore, we hypothesize that the increase in the *ALDH1L1* signal represents unspecific binding. Interestingly, the age of the donor could also play an important role, as we noted for example a much higher background in donor #03 (80 years) in comparison to donors #01 and #02 (19 and 46, respectively). One solution to overcome this problem and to determine the real number of mRNA puncta in tissue sections that have been fixed for an extended period is to use a negative probe on the same section for every experiment, quantify these puncta and subtract these from the mRNA puncta from the mRNA of interest.

Examining additional mRNAs could reveal further insights into their detection dynamics across varying fixation times, potentially related to mRNA stability in the brain itself^27^ or other biological parameters, such as transcript length, sequence or the subcellular localization of the encoded protein. These observations underscore the importance of considering fixation time as a critical factor when employing mRNA detection technologies, as different mRNAs may exhibit distinct responses to prolonged fixation. This observation is specifically problematic in the practical approach, as so far it is difficult to predict how individual mRNA probes react to prolonged fixation. However—with the limited number of donors analysed in this study—the change of the individual mRNA detectability seems to be consistent across different individuals, and therefore, a comparison between different groups, for example a patient cohort and diseased group, might still be feasible despite a non-optimal fixation time, as long as the tissue of the groups has been treated in the same way. Although prolonged antigen retrieval did not result in statistically significant changes in mRNA probes we tested, a clear trend (1/3) towards an increase was observed. This highlights how even minor adjustments to the experimental protocol can influence outcomes, a crucial consideration especially in larger experiments where not all samples can be processed on the same day.

Our findings do neither intend nor allow to conclusively determine an optimal fixation time for post-mortem human brain tissue, as this likely also depends on the mRNA detection methodology employed, the mRNA of interest and probably also on the brain region. However, in line with previous research,^21,22^ our study indicates that specific mRNA detectability generally declines with longer fixation times, suggesting that fixation durations should be kept relatively brief. Already after 5 days of fixation, the normal pattern of oligodendrocyte mRNA distribution was changed, indicating little reliability in the signal. For this reason, a fixation time of 2 days is favourable, although an optimal timepoint needs to be experimentally validated in each scenario. Whilst the specific duration of fixation within an experimental approach may not be critical, it is essential for all tissue blocks within an experimental cohort to undergo uniform treatment to ensure reliable conclusions. In our approach, all tissue blocks were within the same size range, but this variable should also be taken into account when determining the ideal fixation time for an experimental setup. Rather than fixing individual tissue blocks, many brain banks opt to fix the entire hemisphere in formaldehyde, which complicates the exact timing of fixation as the fixative may not uniformly penetrate the tissue. Neutral-buffered formaldehyde is the primary fixative used by most, including brain banks. However, the use of different fixatives could potentially further influence mRNA detectability, as has been demonstrated previously in relation to tissue morphology, immunohistochemistry and RNA quality.^28-30^

Brain banks often store tissue blocks in formalin for extended periods, sometimes without a defined duration, before embedding. As a result, fixation times can vary significantly between individual samples. Unfortunately, this approach renders these tissue blocks unsuitable for mRNA detection. A more suitable approach for brain banks in the future may involve preserving tissue without prior fixation in a snap-frozen state and then fixing small blocks as needed afterwards. This method could maximize the utility of human brain tissue donations for mRNA detection purposes, although other pre- and post-mortem factors such as the health status of the donor or the post-mortem interval should also be considered.

## Conclusion

*In situ* sequencing and other transcriptomic technologies have already substantially contributed to our understanding of pathologies, and cheaper methods and easier accessibility of brain tissue increase the implementation of these methods in many laboratories worldwide. In this study, however, we have clearly demonstrated a complex interplay between mRNA detectability and tissue fixation. The findings underscore the resilience of snap-frozen tissue to freeze-thaw processes and highlight the need for meticulous optimization of fixation conditions in mRNA *in situ* hybridization studies. The observed variability in mRNA responses to fixation time emphasizes the necessity for careful consideration of specific transcripts and experimental objectives when designing protocols for frozen tissue analysis. Furthermore, we strongly recommend to implement a standardized tissue collection and fixation method across different brain banks in order facilitate the collection of brain material for individual researchers and to make results within and across different studies more comparable and meaningful.

## Supplementary Material

fcae430_Supplementary_Data

## Data Availability

The human brain tissue cannot be made available due to ethical reasons, and no code or other scripts have been created in this manuscript.
